# Supplementary material for: Single Cell Transcriptomic and Chromatin Profiles Suggest Layer Vb Is the Only Layer With Shared Excitatory Cell Types in the Medial and Lateral Entorhinal Cortex
Source: Front Neural Circuits. 2022 Jan 26;15:806154. doi: 10.3389/fncir.2021.806154 (PMC8826650; doi:10.3389/fncir.2021.806154)
Supplement: Supplementary file 14 [file Data_Sheet_1.ZIP › MEC_Sup_vs_LEC_Sup_Motifs/homerResults/motif3.similar.html]

motif3

## Information for motif3

C
A
G
T
A
C
T
G
C
T
A
G
C
G
T
A
T
G
C
A
C
T
G
A
C
A
G
T
A
C
T
G
A
G
C
T
T
G
A
C
  
Reverse Opposite:  

A
C
T
G
T
C
G
A
G
T
A
C
G
T
C
A
A
G
C
T
A
C
G
T
G
C
A
T
G
A
T
C
T
G
A
C
G
T
C
A
  

|  |  |
| --- | --- |
| p-value: | 1e-136 |
| log p-value: | -3.140e+02 |
| Information Content per bp: | 1.629 |
| Number of Target Sequences with motif | 7398.0 |
| Percentage of Target Sequences with motif | 35.10% |
| Number of Background Sequences with motif | 3328.1 |
| Percentage of Background Sequences with motif | 27.26% |
| Average Position of motif in Targets | 340.3 +/- 258.9bp |
| Average Position of motif in Background | 330.1 +/- 258.1bp |
| Strand Bias (log2 ratio + to - strand density) | -0.0 |
| Multiplicity (# of sites on avg that occur together) | 1.30 |
| Motif File: | file (matrix) reverse opposite |

### Similar de novo motifs found

|  |  |  |  |  |  |  |  |
| --- | --- | --- | --- | --- | --- | --- | --- |
| Rank | Match Score | Redundant Motif | P-value | log P-value | % of Targets | % of Background | Motif file |
| 1 | 0.883 | A C G T C T A G C T G A A G T C G T C A A C G T A C G T A G C T | 1e-113 | -262.015795 | 22.43% | 16.39% | motif file (matrix) |
| 2 | 0.829 | G C T A T C G A C T G A C G T A A T C G A C G T G T A C G A T C | 1e-91 | -209.584112 | 57.22% | 50.26% | motif file (matrix) |
| 3 | 0.822 | G C A T A C G T C A G T C G T A C G T A C G T A G T C A C G A T C A T G G C A T G A T C G C T A | 1e-88 | -203.097084 | 12.75% | 8.65% | motif file (matrix) |
| 4 | 0.733 | A C G T A C T G A G T C A G T C G T A C A C G T A C G T A C G T | 1e-60 | -138.268542 | 20.40% | 16.11% | motif file (matrix) |
